# The Use of vHIT in the Differential Diagnosis Between Vestibular Migraine and Meniere’s Disease: A Systematic Review and Meta-Analysis

**DOI:** 10.3390/audiolres16010001

**Published:** 2025-12-19

**Authors:** Christos Tsilivigkos, Riccardo Di Micco, Evangelos N. Vitkos, Athanasia Warnecke

**Affiliations:** 1 First Department of Otolaryngology, Hippokration General Hospital, National and Kapodistrian University of Athens, 15772 Athens, Greece; 2 Department of Otorhinolaryngology-Head and Neck Surgery, Hanover Medical School, 30625 Hanover, Germany; dimicco.riccardo@mh-hannover.de (R.D.M.); warnecke.athanasia@mh-hannover.de (A.W.); 3 Department of Oral and Maxillofacial Surgery, Klinikum Dortmund, 44145 Dortmund, Germany; 4 Cluster of Excellence EXC 2177/1 “Hearing4all” of the German Research Foundation, 30625 Hannover, Germany

**Keywords:** vHIT, video head impulse test, vestibular migraine, Meniere’s disease, endolymphatic hydrops

## Abstract

**Background/Objectives:** The diagnosis of vestibular migraine (VM) and Meniere’s disease (MD) is based mainly on clinical criteria. The aim of this study is to systematically review and investigate the potential role of the video Head Impulse Test (vHIT) in the differential diagnosis between VM and MD. **Methods:** A systematic review of the English-language literature was conducted, including studies from database inception to November 2023, in accordance with PRISMA guidelines. Medline (via PubMed), Cochrane Database and Scopus were reviewed. The review included studies involving adult patients diagnosed with VM, MD, or healthy control individuals who underwent vHIT and reported data on vHIT abnormalities, gain, and refixation saccades. The AXIS tool was applied for risk of bias assessment in all cross-sectional studies. A random-effects meta-analysis was performed to compare vHIT gains between individuals with VM and those with MD. **Results:** Eleven cross-sectional observational studies with a case–control comparison design were included, comprising a total of 362 patients with VM, 307 patients with MD, and 135 healthy control subjects. All studies applied the same diagnostic criteria for VM; however, varying criteria were used for the diagnosis of MD. Four studies evaluated the duration of vestibular symptoms, two assessed migraine duration, and six provided a rationale for excluding individuals with overlapping VM and MD diagnoses. Criteria for defining an abnormal vHIT result were specified in six studies. Seven studies reported vHIT gain values for the lateral semicircular canal, while eight presented data on saccade incidence and characteristics. Additionally, four studies were included in the meta-analysis, which yielded a mean difference in the vHIT gain of −0.0203 (95% CI: −0.0789 to 0.0383; *p* = 0.4968), indicating no statistically significant difference between patients with VM and those with MD. **Conclusions:** In this review, vHIT gain did not differ significantly between VM and MD groups, suggesting that vHIT gain alone has limited utility in their differential diagnosis. Combined saccade patterns may still prove clinically useful as more robust and consistent data become available.

## 1. Introduction

Vestibular migraine (VM) and Meniere’s disease (MD) are two episodic vestibular syndromes with overlapping characteristics, making the distinction between them frequently challenging [1]. Meniere’s disease is a peripheral phenomenon connected with endolymphatic hydrops [2], showing a progressive impairment of auditory and peripheral vestibular function on the affected side over the years [3]. Vertigo spells last between 20 min and 12 h and are associated with unilateral sensorineural hearing loss, tinnitus, and aural fullness. Different tests have been implemented in the diagnosis of MD over the last few decades, such as electrocochleography [4], Vestibular Evoked Myogenic Potentials (VEMPs) [5], 3D-FLAIR Magnetic Resonance Imaging (MRI) [6], and multifrequency tympanometry [7]. However, vestibular function asymmetry may not always be detected, even during the acute phase of the disease. Interestingly, MD patients may experience migraine symptoms such as headache, phonophobia, and photophobia in up to 30% of cases [1]. Moreover, more than 20% of MD patients also suffer from migraine [8,9].

Vestibular migraine is thought to be a neuronal disorder arising from combined neurological and vascular events [10]. The cortical spreading depression of a migraine attack, combined with the activation of the trigeminovascular system, which innervates the labyrinthine vessels, can result in modifications of the vascular flow in the peripheral vestibular organs, potentially explaining the vestibular deficit in patients [11]. Cortical spreading depression (CSD) occurs in the gray matter, initiates within the occipital cortex, and can spread to other areas, generating a wave of neural hyperexcitability followed by long-lasting neural suppression [12]. It is believed to influence the vestibular cortex and the brainstem [13], leading to impaired peripheral vestibular function [14].

Although vestibular function in VM is normal during symptom-free intervals [15], patients may present with spontaneous or positional vertigo, unsteadiness, and balance problems lasting from minutes to several days, either simultaneously with or following headache attacks [16,17]. The recording of spontaneous or positional nystagmus may be evident during acute attacks, but rapidly disappears [18,19]. The diagnosis of VM does not require auditory symptoms; however, up to 38% of patients exhibit an auditory aura with tinnitus during vertigo spells [20], making differentiation from MD particularly difficult.

Due to these highly heterogeneous vestibular, audiological, and neurological findings, the diagnosis of both VM and MD is currently based on clinical presentation over an extended period [21,22]. There is no consensus on which vestibular tests can reliably differentiate between the two diseases. Recently, there has been growing interest in the application of the video head impulse test (vHIT) for assessing the vestibulo-ocular reflex (VOR) in episodic vestibular syndromes [23].

Since VM and MD patients may be otologically and neurologically asymptomatic during examination, vHIT could provide a means to characterize and differentiate between the two diseases more efficiently. However, results to date have been controversial. This review aimed to systematically summarize the use of vHIT in the differential diagnosis of patients with MD, VM, and healthy subjects based on the available literature, and to perform a meta-analysis of vHIT gain differences between patients with VM and MD.

## 2. Materials and Methods

### 2.1. Study Design

The current review was not registered in a systematic review registry. To form this research question, a systematic review of the literature and meta-analysis according to the PRISMA (Preferred Reporting Items for Systematic Reviews and Meta-Analyses) statement was conducted [24]. All studies published from database inception to November 2023 were included. We addressed our research question with the use of PICO (Population/Participants, Intervention, Comparison, Outcome) structure:

Population/Participants: (i) Adult (>18 years old) patients with diagnosed (according to the MD criteria) MD; (ii) adult patients with diagnosed (according to the VM criteria) VM; and (iii) adult control subjects.

Intervention: vHIT in patients with MD and VM in the interictal phase, and in healthy subjects

Comparison: Comparisons of vHIT outcomes were made between affected and unaffected ears in MD patients; between right and left (or both) ears in VM patients; and between right and left ears in healthy controls.

Outcomes: The primary outcomes evaluated are (i) the rate of abnormal vHIT results, (ii) vHIT gain, and (iii) saccades (overt and covert).

### 2.2. Eligibility Criteria

Research articles published in English that directly compared vHIT outcomes in adults diagnosed with MD and/or VM, as well as healthy control subjects, were included. All ears included had to be free of middle ear pathology and have an intact tympanic membrane.

The following exclusion criteria were applied: (i) studies published in languages other than English; (ii) case reports and case series; (iii) systematic reviews of interventions and meta-analyses; (iv) theses; (v) studies with fewer than five reported patients per group; (vi) studies involving only unrelated patient groups (e.g., patients with other middle or inner ear diseases); (vii) studies including patients during acute episodes of MD or VM; (viii) studies involving children; (ix) studies with unavailable relevant patient data; (x) studies that did not report at least one relevant outcome measure.

It was agreed a priori that if multiple studies reported data from the same population, only the study with the best design would be included in the meta-analysis. However, our systematic review would encompass studies with overlapping populations if they met the inclusion criteria. No publication year restrictions were applied.

### 2.3. Search Strategy

We searched Medline via PubMed with the following research strategy:

(“migraine associated vertigo” OR “vestibular migraine”) AND (“video head impulse test”).

Additionally, we searched the Cochrane Database with the following algorithm:

(“vestibular migraine”) AND (“video head impulse test”),

and Scopus using:

((TITLE-ABS-KEY (“video head impulse test”))) AND ((TITLE-ABS-KEY (“vestibular migraine”)))

The search date was 26 November 2023.

Two independent reviewers (CT and RDM) screened the search results based on titles and abstracts to assess their eligibility. Selected articles then underwent a full-text evaluation to determine their final inclusion. In cases of disagreement between the reviewers, consensus was reached through discussion with the senior author (AW). Furthermore, a manual search of the reference sections of included articles was conducted to identify any additional potentially eligible studies [25].

### 2.4. Data Extraction and Tabulation

The first two authors (CT and RWM) independently extracted data from the included articles using a standardized, pre-designed template for evidence collection. Any disagreements were resolved through discussion with the senior author (AW). The extracted data included: (i) study characteristics (authors, year, country, study design), total patient count, and the number of VM, MD, and control patients, categorization of ears as right/left, affected/unaffected; (ii) patients’ baseline characteristics (age and sex); (iii) vHIT outcomes (abnormal vHIT, vHIT gain, overt and covert saccades incidence, saccade velocity, saccade interval, and saccade time); (iv) diagnostic criteria for VM and MD; (v) rest of the otological examination; (vi) the definition of abnormal vHIT and the passive lateral head impulses performed across the different studies; (vii) the duration of vestibular symptoms, and of migraine headaches; and (viii) the description of exclusion of a dual diagnosis of VM and MD. Only data available in the original articles were used.

### 2.5. Quality of Evidence Assessment

All studies included in our systematic review and meta-analysis were cross-sectional ones. The quality of the articles included in our systematic review and meta-analysis was assessed using the AXIS tool for cross-sectional studies [26]. Two independent researchers (CT and RDM) applied the tool to all studies included in the systematic review and examined the 20 domains that the tool addresses. A third-party reviewer arbitrated if the two researchers disagreed. The results of the bias assessment can be found in the Supplemental Material Table S1. Overall, most studies demonstrated a moderate risk of bias, primarily due to the lack of sample size justification and insufficient measures to address non-responders. Furthermore, participant selection was often not justified as representative of the target population, response rates were generally unreported, and study limitations were inadequately described.

### 2.6. Statistical Analysis

A meta-analysis was conducted to compare vHIT gain between patients with VM and MD. Reporting of vHIT gain is not consistent across studies, as some authors present data per ear (affected vs. unaffected in MD; right vs. left in VM), while others report per-patient values. To ensure comparability in our meta-analysis, we used only the affected ear in patients with MD, whereas for VM, we calculated the mean gain from both ears for each patient.

Random-effect models were employed to calculate the overall mean difference between the two groups, accounting for between-study variability. Heterogeneity was assessed using the I^2^ statistic to quantify inconsistency across studies, and Cochran’s Q test was applied to evaluate statistical significance. Effect sizes were presented with corresponding 95% confidence intervals (CIs), and a forest plot was generated to illustrate individual study estimates and the pooled effect size. Statistical significance was determined by the *p*-value, with the directionality of effects labeled to indicate whether the findings favored VM or MD. All analyses were performed using the ‘meta’ package in R (version 4.4.1).

## 3. Results

### 3.1. Study Selection and Baseline Characteristics

**Figure 1:**
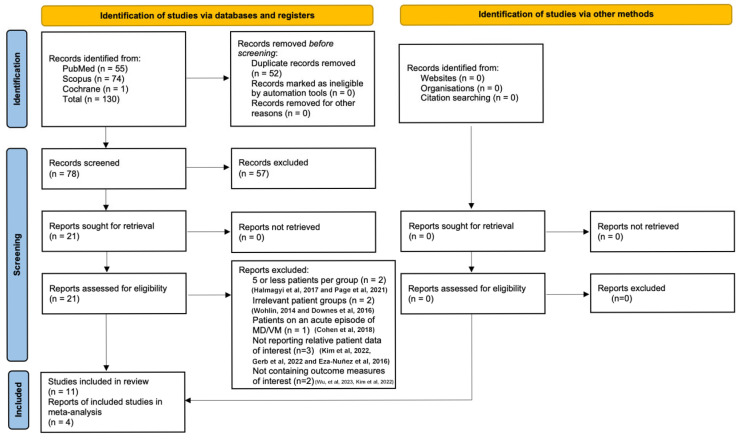
PRISMA 2020 flow diagram for new systematic reviews, which included searches of databases, registers and other sources [23,24,25,26,27,28,29,30,31,32].

The literature search is presented in the PRISMA flowchart (Figure 1). After performing a systematic literature search, we retrieved a total of 130 studies. Subsequently, we excluded 52 duplicate records, and in the remaining 78 studies, we conducted title and abstract scanning. Fifty-seven studies were excluded, leading to a total of 21 articles. Through full-text review, we excluded another 10 articles. Two of them reported 5 or fewer patients per group [27,28], two included irrelevant patient groups [29,30], one reported patients with MD or VM during an acute episode [31], three did not report the relative patient data of interest [32,33,34], and two did not contain at least one outcome measure of interest [35,36]. The manual search of the references section of the retrieved articles did not conclude with any additional articles. Overall, 11 studies were included in this systematic review. Among these, five studies were prospective cross-sectional observational studies with a case–control comparison design and four were retrospective cross-sectional observational studies with a case–control comparison design. The remaining two studies had similar designs, although the manuscript did not clearly indicate whether they were conducted prospectively or retrospectively. In order to assess geographical contributions, we reviewed the studies by their country of origin. Four of these studies were performed in Turkey [14,37,38,39], three in China [40,41,42], two in Egypt [43,44], one in Germany [1], and one in Italy [45]. The baseline characteristics of the included articles and the reported patients are highlighted in Table 1.

### 3.2. Patients’ Cohort

Ultimately, our systematic review included 11 studies, encompassing a total of 362 VM patients, 307 MD patients, and 135 control subjects. Two studies compared patients with definite VM to those with definite MD and healthy controls [38,39], four studies compared patients with definite VM to those with MD [37,41,42,45], while four studies directly compared individuals with definite VM to healthy controls [14,40,43,44]. One study compared patients with definite or probable VM to patients with definite or probable MD [1]. In four of these studies, additional groups were included: probable VM [40], unilateral vestibular dysfunction, Ramsay–Hunt syndrome, bilateral vestibular hypofunction, benign paroxysmal positional vertigo, acoustic neuroma [42], recurrent vestibulopathy [39], and patients with migraine but not VM symptoms [14].

Six studies reported vHIT results in MD patients by comparing the affected to the healthy unaffected ears [1,37,38,39,41,42]. In only one study presented MD vHIT results by the affected side (right vs. left) [45]. For VM, seven studies provided separate right-ear and left-ear vHIT results [1,38,39,40,41,43,45], whereas four studies did not differentiate by the measured sides [14,37,42,44].

In all 11 studies, the same diagnostic procedure for VM was used, namely the consensus developed by the Committee for Classification of Vestibular Disorders of the Bárány Society and the Migraine Classification Subcommittee of the International Headache Society (IHS) [21]. Yollu et al. applied the criteria from the Headache Classification Committee of the IHS, which incorporates the aforementioned consensus [14]. In contrast, different diagnostic criteria were used in the seven studies involving patients with MD. Specifically, four studies used the 2015 international consensus diagnostic criteria for MD [38,39,41,45], proposed by the different societies [22], whereas three studies followed the earlier (1995) guidelines [1,37,42] proposed by the AAO-HNS [46].

All 11 studies included vHIT as part of their diagnostic approach. However, each study also incorporated additional tests in its diagnostic battery. The study groups, the diagnostic criteria used for VM and MD, and the otological examinations performed in the included studies, are presented in Table 1.

The duration of vestibular symptoms was provided by four research teams. In a study by Balayeva et al., the VM group had vestibular symptoms for 79.2 ± 49.2 months, while the MD group had symptoms for 75.6 ± 54 months [38]. Blödow et al. reported that in the VM group, 10 patients had early disease (<5 years), and 13 had advanced disease (>5 years). In the MD group, 17 patients had early disease, and 13 had advanced disease [1]. Du et al. provided data on the duration of symptoms in the VM group, with a range from 1 month to 10 years [40]. Kaçan et al. reported a mean duration of 5.2 years for patients with VM and 5.6 years for those with MD [39].

The duration of migraine headaches was described in two studies. According to ElSherif et al., 72% of patients had migraine symptoms lasting less than 5 years, while 28% had migraines lasting over 5 years [44]. Martines et al. reported that 57.14% of the patients experienced migraines before the age of 25, with a mean age of onset at 25.14 ± 10.03 years [45].

The possibility of a dual diagnosis of VM and MD was considered and described in the manuscripts of six studies [14,37,41,42,44,45]. According to Du et al., individuals with both migraine symptoms and MD in both ears were excluded [41]. Yilmaz et al. aimed to exclude patients with concurrent MD in the VM group by not including those with hearing thresholds >20 dB in the lower frequencies [37].

### 3.3. vHIT Outcomes

Six studies cited the conditions under which vHIT results were considered abnormal [1,14,37,42,43,45]. These conditions, as well as the passive lateral head impulses during vHIT are presented in Table 2. Overall, only 3 out of the 11 studies provide data (either regarding gain or saccades) from the six semicircular canals [14,40,43].

### 3.4. vHIT Gain Outcomes

Seven studies provided data regarding vHIT gain for the lateral semicircular canals in subjects with VM, MD, and healthy controls [1,38,39,40,42,43,45]. The authors in two studies have assessed gain in all semicircular canals [14,43]. Moreover, an abnormal vHIT gain rate was assessed in two studies [14,42], and gain asymmetry between the two sides in two studies [14,37].

According to Balayeva et al., the vHIT gain of the lateral semicircular canal in the affected ears of patients with MD was significantly lower compared to those with VM (*p* = 0.003) and healthy controls (*p* < 0.001) [38]. Similarly, Blödow et al. found a statistically significant difference in the incidence of lateral canal vHIT gain abnormalities between MD (37%) and VM patients (9%) (*p* = 0.025) [1]. In contrast, Du et al. reported an abnormal vHIT gain in 10% of MD patients and 9% of VM patients, with no significant difference between affected and unaffected ears in the MD group (*p* > 0.05) [42]. Martines et al. also observed no significant difference in vHIT gain between VM and MD groups (*p* = 0.76) [45].

Furthermore, Du et al. found no statistically significant difference between the left and right ears in VM patients (*p* = 0.066) [40]. ElSherif et al. reported slightly higher vHIT gains on the right side for both the lateral and anterior canals in VM and healthy individuals, though without statistical significance. However, vHIT gains in the lateral and posterior canals were non-significantly lower in VM patients compared to controls, while the right posterior canal exhibited a statistically significant reduction in the VM group [43]. Kaçan et al. reported a significant difference in vHIT gain between affected and unaffected ears in MD patients (*p* = 0.015), whereas no such difference was observed in VM or healthy individuals. Moreover, when comparing VM, MD, and control groups, no significant differences were found in overall vHIT gain values (*p* = 0.53) [39].

The above data are presented in Table 3.

### 3.5. Saccade Outcomes

The incidence of saccadic movements in patients with VM, MD, and healthy controls was investigated in eight studies [14,37,39,40,41,42,43,44]. In two of these studies, the saccades were further classified as overt and covert [14,37]. Data regarding saccade velocity in these groups were provided on three occasions [40,41,43], regarding saccade latency in two studies [40,43], and regarding saccade time in one study [41]. Saccadic movements in the vertical semicircular canals were assessed in two studies [40,43]. Only two studies mentioned the recording of second and third saccades, but considered only the first saccade as clinically significant [40,41]. Saccade divergence was evaluated in one study using the Perez and Rey (PR) score [42].

Du et al. reported a high incidence of saccades in the lateral semicircular canals (86%) among VM patients, with no significant differences in saccade velocity or latency between the right and left ears [40]. In contrast, another study found that patients with MD exhibited more significant interaural differences compared to those with VM, specifically higher saccade velocity (*p* = 0.00), earlier onset (*p* = 0.01), and greater temporal clustering (*p* = 0.003) on the affected side [41]. Moreover, both VM and MD patients demonstrated a higher frequency of saccades than abnormal vHIT gain. In patients with MD, no statistically significant difference in PR was observed between the affected and unaffected ears (*p* = 0.20) [42].

ElSherif et al. noted a higher frequency of refixation saccades in VM patients compared to healthy controls [43], while another study found that 36% of VM patients exhibited refixation saccades, compared to only 10% in the control group [44]. Kaçan et al. observed saccades in 15% of MD patients and 5% of those with VM [39]. Yilmaz et al. reported a statistically significant difference in the incidence of saccades between MD (37.3%) and VM patients (10%) (*p* < 0.001) [37]. Lastly, Yollu et al. found that VM patients demonstrated a significantly higher rate of overt saccades compared to healthy individuals (*p* = 0.008), though the incidence of covert saccades was not statistically significant (*p* = 0.333) [14].

Data regarding saccade parameters are presented in Table 4.

### 3.6. Meta-Analysis

**Figure 2:**
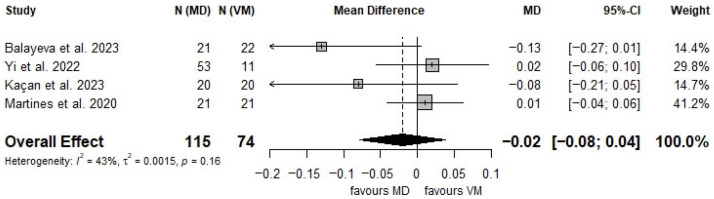
Forest plot comparing vHIT gain between the MD and the VM groups [38,39,42,45].

A total of 4 studies reporting on 189 patients (115 MD and 74 VM) were included in the meta-analysis [38,39,42,45]. The analysis was conducted using the inverse variance method. The random-effects model yielded a mean difference in vHIT gain of −0.0203 (95% CI: −0.0789 to 0.0383; *p* = 0.4968) (Figure 2), indicating no statistically significant difference between patients with VM and MD.

Heterogeneity across studies was moderate, with an I^2^ value of 42.7% (95% CI: 0.0% to 80.8%). The estimated between-study variance (τ^2^) was 0.0015 (95% CI: 0.0000 to 0.0697), with a τ value of 0.0385 (95% CI: 0.0000 to 0.2639), reflecting moderate variability in effect sizes across studies.

## 4. Discussion

The vHIT is a simple, high-speed video system designed to detect peripheral vestibular lesions [47]. It has become a widely used diagnostic tool in vertigo and dizziness clinics worldwide [23]. Video Head Impulse Test represents an evolution of the classical Head Impulse Test (HIT), a bedside clinical test traditionally used to identify overt saccades and, by extension, peripheral vestibular deficits [47]. Unlike the HIT, the vHIT provides quantitative data by measuring eye velocity and analyzing the VOR and both covert and overt saccades in response to small, rapid, and unpredictable head impulses in the horizontal and vertical planes [47].

Differentiating between VM and MD can be challenging due to their overlapping symptoms, including episodic vertigo, nausea, and imbalance. Both conditions are prevalent, and diagnosis largely relies on clinical history. In MD, pure-tone audiometry is also employed as a diagnostic criterion according to international guidelines [21,22].

While the pathophysiology of MD remains partly unclear, it is generally regarded as a peripheral vestibular disorder affecting the membranous labyrinth. In contrast, VM is associated primarily with central vestibular dysfunction [1]. Given these differing pathologies, it is logical to pursue diagnostic tools capable of distinguishing between central and peripheral vestibular lesions. Vestibular testing and imaging modalities such as MRI play an important role in this diagnostic process. Among these, vHIT has shown promising potential for clinical differentiation in some studies.

Traditionally, caloric irrigation has been the gold standard for assessing peripheral vestibular disorders due to its high sensitivity during the static decompensation phase. However, caloric testing has limited utility in evaluating dynamic vestibular compensation after a peripheral lesion. In contrast, vHIT offers valuable insight into dynamic compensation by detecting both overt and covert saccades. Notably, the presence of covert saccades may serve as an indicator of the dynamic compensation status [48].

The study of the VOR is based on three neuronal arcs that connect the vestibular organs to the vestibular nuclei in the brainstem, the brainstem to the oculomotor nuclei, and the oculomotor nuclei to the extraocular muscles [49]. Considering its natural high frequency of 3–6 Hz, vHIT offers a more physiological and patient-tolerable method to analyze the VOR in episodic vestibular syndromes compared to traditional caloric tests, which have a frequency of 0.003 Hz, and other formal vestibular studies [50]. However, the data acquired through vHIT is complex and must be analyzed in terms of both VOR gain and refixation saccades.

When analyzing the VOR gain (the ratio between eye and head velocity), vHIT studies on MD and VM are controversial. vHIT gain loss has been reported in approximately 10–55% of MD patients and 9–18% of VM patients (when compared to healthy subjects) [1,37,40]. Generally, MD patients tend to show more frequent vHIT gain loss than VM patients; however, there are reports of completely normal vHIT gain values for both MD and VM [51]. The short duration of the disease may be the underlying reason for normal results [51].

A point of debate is the timing of vHIT recordings. Often, vHIT is performed during the interictal phase [45], and there is no consistent information on the duration of the disease, leaving it unclear as to whether normal vHIT results in MD or VM are overestimated [1]. The vHIT is known for being highly specific but variably sensitive, largely depending on the acuteness of the vestibular disorder and the degree of canal paresis [52,53].

Another challenge lies in the poor correlation between vHIT and caloric test results, despite both tests tending to be abnormal more often in individuals with MD than in those with VM [45]. Caloric test abnormalities of the horizontal canal VOR are reported in 42–76% of MD patients, compared to 7–34% of VM patients, underscoring the higher sensitivity of caloric testing in detecting horizontal canal VOR hypofunction in both conditions [20,37]. This discrepancy may relate to how endolymphatic hydrops differentially affects regular and irregular vestibular afferents. Hydrops is thought to initially impair the peripheral zone of the crista ampullaris, where type II hair cells and regular afferents predominate. Because these afferents respond best to low-frequency stimulation, which is precisely what caloric testing evaluates, early pathology in this region can produce abnormal caloric results. In contrast, the central crista, composed mainly of type I hair cells and high-frequency irregular afferents, is assessed by the vHIT. This region may become involved later in the course of MD, which could explain why vHIT abnormalities typically appear later than caloric deficits [54].

Overall, according to Blödow et al., calorics were pathological in 47% of the patients, compared to vHIT, which was abnormal in 25% of cases. The sensitivity of combining caloric testing and vHIT was estimated at 52% for MD and 40% for VM. Interestingly, only 19% of patients with MD and VM exhibited abnormalities in both vHIT and caloric tests, while 47% demonstrated normal results in both tests [1]. In a study by Yilmaz et al., vHIT was found to be less sensitive than calorics in assessing VOR deficits. They concluded that vHIT should not replace calorics, but should be used as an adjunct test in the diagnostic battery [37].

Once a vHIT gain anomaly is identified, it remains challenging to differentiate between VM and MD. Some studies have found a tendency for lower vHIT gain values on the affected side in long-standing MD patients (over 6 years) compared to VM patients [38], while other studies did not find any statistically significant difference between the vHIT results in the two conditions [39,45]. As a result, horizontal vHIT gain loss alone may not be sufficient to differentiate between VM and MD, and additional vHIT parameters must be considered. Additionally, our meta-analysis, although including only 4 studies and 189 overall patients, did not find a statistically significant difference in vHIT gain between patients with VM and MD (*p* = 0.4968).

The brainstem compensates for gaze or prediction errors occurring due to peripheral vestibular impairment by generating eye saccades, which are detected by vHIT without influencing the VOR gain. These compensatory movements occur either during head movement (covert saccades) or after head movement has stopped (overt saccades) across all vestibular canals, and the time to correct gaze or prediction error is measured. As with vHIT gain, saccade velocity can indicate peripheral vestibular impairment [55]. Saccades may also be generated by positional errors [56], combined oculomotor and cervical proprioception input [57], and progressive aging of the vestibular system [58], which explains their occasional presence even in healthy individuals.

In VM patients, the rate of bilateral refixation saccades increases within one week of a vertigo spell, compared to healthy subjects. This occurs even in the presence of normal or low vHIT gain [43], making refixation saccades more sensitive to peripheral vestibular deficits in VM than vHIT gain. The presence of low-amplitude covert saccades, despite normal vHIT gain, represents the correction of eye drifts toward the neutral position. Covert saccades in VM can be explained by a disturbance in the spontaneous firing rate at the level of the vestibular nuclei [14]. This can be demonstrated through magnetic resonance spectroscopy using lactate peaks in the tissue due to CSD in the occipital lobe spreading to the brainstem [44]. Refixation saccades are also present in MD patients but are more clustered on the affected side [1,37].

We chose not to perform a meta-analysis of saccade occurrence in VM and MD due to substantial heterogeneity in how the included studies reported saccadic events. In particular, many studies did not clearly specify whether their outcomes referred to overt, covert, or total saccades.

A more in-depth analysis of the raw recorded saccades from vHIT could help in phenotypically characterizing VM and MD. A recently proposed saccade time variation parameter, the PR score, indicates the vestibular recovery status [59], but it ignores unapparent saccades (those with velocities under 65°/s). Analysis of raw saccade data in VM patients has revealed that 86% of VM patients with normal vHIT gain exhibit small, scattered, bilateral saccades, compared to the rare, randomly distributed unilateral saccades in 29% of healthy controls and MD patients [40]. The small amplitude of these scattered saccades and the generally normal vHIT gain suggest that the peripheral vestibular function remains largely intact but is subtly and permanently impaired.

In contrast, MD patients demonstrate saccades with higher velocity, earlier onset, and more time-domain clustering on the affected side [41]. According to Du et al., MD patients typically show normal gain values on both sides, but the saccade recording rate is equally distributed on both sides, with PR scores ranging from scattered to gathered [42]. Although raw saccade data remain understudied, initial results provide a promising way to differentiate VM and MD phenotypically using vHIT. Further research is needed to confirm these findings.

This systematic review and meta-analysis have several limitations. Firstly, all studies had observational cross-sectional designs, and there were no cohorts assessing patients over time. Therefore, the results of this meta-analysis should be interpreted with caution, as numerous confounding factors exist. This can be seen in the bias assessment of the included studies, which was conducted with the AXIS tool for cross-sectional studies (Supplemental Material Table S1). Secondly, while 11 studies were included, the data were not homogeneous, and the sample sizes were small, making it difficult to conduct a robust meta-analysis. Thirdly, the heterogeneity of vHIT gain across studies was moderate. This heterogeneity may also reflect the inconsistent reporting of vHIT gain values across studies. Fourth, as previously noted, this review was not registered in a systematic review registry. This introduces a potential risk of selective reporting and methodological bias. However, the primary reason for not registering was that, at the time this study commenced, the PROSPERO database did not accept submissions of systematic reviews on diagnostic test accuracy. Other limitations include the variation in diagnostic criteria for MD, differences in study groups, inconsistent vHIT outcome analyses, variability in the conduct of vHIT measurements, and the restriction of our methodology to studies published in English. Despite these limitations, this study provides the most reliable evidence available to date.

In conclusion, the diagnosis of VM and MD continues to rely primarily on clinical criteria. Current evidence suggests that the vHIT cannot distinguish between the two conditions, despite vHIT gain loss being observed more frequently in MD patients than in those with VM. The diagnostic potential of saccade patterns on vHIT remains insufficiently studied and inconclusive. Caloric testing continues to be the gold standard vestibular assessment for differentiating VM from MD.

## Figures and Tables

**Table 1:** Study and patient characteristics of the studies included in the systematic review.

Author	Study Design	Total Patient Count	Study Groups	VM/MD/Control Patients and Age	Diagnostic Criteria	Otological Examination
Balayeva F et al. 2023 [38]	Prospective observational, cross-sectional	64	Definite VM vs. definite MD vs. HC	22 (47.2 ± 8.9 y.o.)/21 (50.1 ± 9.3 y.o.)/21 (45.6 ± 7.5 y.o.)	VM: consensus of the Bárány Society and the IHC MD: joint formulation by the Classification Committee of the Bárány Society, JSER, EAONO, AAO-HNS and KBS	PTA, vHIT, fHIT, cVEMP
Blödow A et al. 2014 [1]	Retrospective, multi-center, observational, cross-sectional	53	Definite MD or probable MD vs. definite VM or probable VM	23 (46 ± 13 y.o.)/30 (54 ± 12 y.o.)/N/A	VM: consensus of the Bárány Society and the IHC MD: 1995 criteria of the AAO- HNS	standard ENT/neurological examination, PTA, calorics, vHIT
Du Yi et al. 2022 [40]	Retrospective observational, cross-sectional	92	VM vs. probable VM vs. HC	14 (44.21 ± 11.93 y.o.)/N/A/14 (35.36 ± 4.30 y.o.)	VM: consensus of the Bárány Society and the IHC	neurotological examination, vHIT, calorics
Du Yi et al. 2023 [41]	Retrospective observational, cross-sectional	178	Definite VM vs. definite MD	75 (47.24 ± 13.88 y.o.)/103 (51.11 ± 12.34 y.o.)/N/A	VM: consensus of the Bárány Society and the IHC MD: joint formulation by the Classification Committee of the Bárány Society, JSER, EAONO, AAO-HNS and KBS	vHIT
Du Yi et al. 2021 [42]	Retrospective observational, cross-sectional	165	VM vs. MD vs. UVD vs. RHS vs. BVH vs. BPPV vs. AN	11 (41.5 ± 9.1 y.o.)/53 (51.9 ± 14.0 y.o.)/N/A	VM: consensus of the Bárány Society and the IHC MD: 1995 criteria of the AAO- HNS	history, medical records, imaging results, neurotological examination, PTA, vHIT, calorics
ElSherif M et al. 2018 [43]	Observational, cross-sectional	120	Definite VM vs. HC	80 (39.06 ± 7.43 y.o.)/N/A/40 (36.95 ± 6.65 y.o.)	VM: consensus of the Bárány Society and the IHC	history, PTA, vHIT, videonystagmography
ElSherif M et al. 2020 [44]	Observational, cross-sectional	45	VM vs. HC	25 (41.64 ± 8.74 y.o.)/N/A/20 (42.75 ± 8.27 y.o.)	VM: consensus of the Bárány Society and the IHC	history, PTA, vHIT, videonystagmography, MRI and proton magnetic resonance spectroscopy
Kaçan M et al. 2023 [39]	Prospective observational, cross-sectional	78	VM vs. MD vs. HC vs. recurrent vestibulopathy	20 (48.6 ± 8.6 y.o.)/20 (52.4 ± 5.1 y.o.)/20 (51.4 ± 9.7)	VM: consensus of the Bárány Society and the IHC MD: joint formulation by the Classification Committee of the Bárány Society, JSER, EAONO, AAO-HNS and KBS	PTA, vHIT, fHIT, cVEMP
Martines F et al. 2020 [45]	Prospective observational, cross-sectional	42	Definite VM vs. definite MD	21 (43.61 ± 12.31 y.o.)/21 (43.28 ± 7.3 y.o.)/N/A	VM: consensus of the Bárány Society and the IHC MD: joint formulation by the Classification Committee of the Bárány Society, JSER, EAONO, AAO-HNS and KBS	Otological, neurological examination, questionnaire about VM, PTA, vHIT, EcochG
Yilmaz MS et al. 2021 [37]	Prospective observational, cross-sectional	109	Definite VM vs. definite unilateral MD	50 (44.86 ± 9.36 y.o.)/59 (48.37 ± 11.66 y.o.)/N/A	VM: consensus of the Bárány Society and the IHC MD: 1995 criteria of the AAO-HNS	history, otological, neurological examination, PTA, calorics, vHIT
Yollu U et al. 2017 [14]	Prospective observational, cross-sectional	61	VM vs. HC vs. migraine-only pts	21 (36.0 ± 10.9 y.o.)/N/A/20	VM: consensus of the Bárány Society and the IHC (later included in the beta version of the third edition of the ICHD guideline)	sociodemographical data form, PTA, speech audiometry, vHIT, dynamic posturography, EcochG

Abbreviations: AAO-HNS: American Academy of Otolaryngology—Head and Neck Surgery; AN: acoustic neuroma; BPPV: benign paroxysmal positional vertigo; BVH: bilateral vestibular hypofunction; cVEMP: cervical vestibular evoked myogenic potential; EAONO: European Academy of Otology and Neurotology; EcochG: electrocochleography; fHIT: functional head impulse test; HC: healthy controls; ICHD: International Classification of Headache Disorders; IHC: International Headache Society; JSER: Japan Society for Equilibrium Research; KBS: Korean Balance Society; MRI: Magnetic Resonance Imaging; N/A: not applying; pts: patients; PTA: pure tone audiometry; RHS: Ramsay–Hunt syndrome; y.o.: years old; UVD: unilateral vestibular dysfunction.

**Table 2:** Definition of passive lateral head impulses and abnormal vHIT results.

Authors	Passive Lateral Head Impulses	Abnormal vHIT
Balayeva F et al. 2023 [38]	Peak head velocities between 150 and 250°/s, amplitude 10–20°	Not analyzed
Blödow A et al. 2014 [1]	Peak velocity 200°/s, amplitude 15–20°, duration 150–200 ms	When both of the following criteria are satisfied: 1. Gain < 0.8 2. Presence of refixation saccades within 700 ms after the initiation of a head impulse
Du Yi et al. 2022 [40]	Peak velocity > 150°/s	Not analyzed
Du Yi et al. 2023 [41]	Peak velocity > 150°/s	Not analyzed
Du Yi et al. 2021 [42]	Not analyzed	Abnormal vHIT gain when <0.8
ElSherif M et al. 2018 [43]	Peak velocity 200°/s, amplitude 10–20°	Abnormal vHIT gain when <0.8 for the lateral canals and <0.7 for the vertical canals
ElSherif M et al. 2020 [44]	Peak velocity 100–200°/s, amplitude 10–20°	Not analyzed
Kaçan M et al. 2023 [39]	Peak head velocity 150–250°/s, amplitude 10–20°	Not analyzed
Martines F et al. 2020 [45]	Peak velocity 200°/s	Abnormal lateral canal function when: 1. mean gain < 0.8 and gain asymmetry > 5% 2. identification of refixation saccades
Yilmaz MS et al. 2021 [37]	Not analyzed	Abnormal lateral canal function when: 1. gain < 0.8 2. identification of overt and covert saccades
Yollu U et al. 2017 [14]	Not analyzed	Normal gain values were considered: 0.8–1.2 for the lateral and 0.7–1.2 for the vertical canals

**Table 3:** VOR gain outcomes and additional information.

Author	VOR Gain	Additional Information
Balayeva F et al. 2023 [38]	VM: 0.95 (0.27–1.28) *	-
	0.94 (0.27–1.21) (right) *	
	0.95 (0.28–1.28) (left) *	
	MD: 0.81 (0.24–1.06) (AE) *	
	0.9 (0.3–1.1) (UE) *	
	HC: 1 (0.76–1.35) *	
	(0.76–1.18) (right) *	
	1 (0.88–1.35) (left) *	
Blödow A et al. 2014 [1]	MD: 0.83 ± 0.2 (AE) **	-
	0.95 ± 0.16 (UE) **	
Du Yi et al. 2022 [40]	VM: 0.97 ± 0.08 (right) **	-
	0.92 ± 0.07 (left) **	
Du Yi et al. 2023 [41]	-	-
Du Yi et al. 2021 [42]	VM: 0.93 ± 011 **	Abnormal VHIT gain rate (estimated on both ears) VM: 9% MD: 10%
	MD: 0.95 ± 0.15 (AE) **	
ElSherif M et al. 2018 [43]	VM: 0.9 (0.4–1.8) (right) *	-VM pts: 51.3% (41/80) sufferers of migraine with aura, 37.5% (30/80) migraine only, 11.3% (9/80) chronic migraine -VM gains: LA 1.1 ± 0.3, LP 1.2 ± 0.3, RA 1.2 ± 0.3, RP 0.98 ± 0.25 HC gains: LA 1.2 ± 0.3, LP 1.2 ± 0.3, RA 1.3 ± 0.3, RP 1.1 ± 0.27
	0.87 (0.5–1.1) (left) *	
	0.9 ± 0.3 (right) **	
	0.84 ± 0.13 (left) **	
	HC: 0.9 (0.4–1.8) (right) *	
	0.89 (0.5–1.1) (left) *	
	1 ± 0.3 (right) **	
	0.89 ± 0.15 (left) **	
ElSherif M et al. 2020 [44]	-	-All VM pts had migraines with aura
Kaçan M et al. 2023 [39]	VM: 0.97 (0.37–1.21) *	-
	0.98 (0.43–1.21) (right) *	
	0.97 (0.37–1.2) (left) *	
	MD: 0.92 (0.24–1.10) (AE) *	
	0.98 (0.81–1.11) (UE) *	
	HC: 1.01 (0.84–1.2) *	
	1.01 (0.88–1.13) (right) *	
	1.08 (0.84–1.2) (left) *	
Martines F et al. 2020 [45]	VM: 0.93 ± 0.06 (right) **	-
	0.95 ± 0.09 (left) **	
	MD: 0.95 ± 0.16 (right) **	
	0.90 ± 0.08 (left) **	
Yilmaz MS et al. 2021 [37]	-	-Abnormal vHIT
		VM: 18% (9/50)
		MD: 39% (23/59) (AE)
		-Gain asymmetry
		VM: 24% (12/50)
		MD: 35.6% (21/59) (AE)
Yollu U et al. 2017 [14]	-	-Abnormal gain in at least 1 canal
		VM: 28.6% (6/21)
		HC: 15% (3/20)
		(All the pts above had an abnormal gain in only 1 canal)
		-Asymmetry in any canal
		VM: 42.9% (9/21)
		HC: 15% (3/20)
		(8 out of these 9 pts had asymmetry in 1 canal and 1 pt has asymmetry in 2 canals. The 3 HCs has asymmetry in 1 canal)

Abbreviations: AE: affected ears; HC: healthy controls; LA: left anterior semicircular canal; LP: left posterior semicircular canal; pts: patients; RA: right anterior semicircular canal; RP: right posterior semicircular canal; UE: unaffected ears. * median and range. ** mean and standard deviation.

**Table 4:** Characteristics of the saccades in vHIT in VM patients, MD patients, and healthy subjects in the different studies.

	Saccade Incidence	Saccade Velocity	Saccade Latency **	Saccade Time
Du Yi et al. 2022 [40]	VM: 12/14–86%	VM: 72.77 ± 32.06°/s (L), 78.74 ± 30.99°/s (R)	VM: 288.90 ± 61.74 ms (L), 279.65 ± 70.01 ms (R)/ HC: 152–524 ms	
Du Yi et al. 2023 [41]	VM: 58/75–77% (L), 57/75–76% (R)/	VM: 67.12 ± 26.13°/s (L), 72.70 ± 31.06°/s (R)		VM: 105.62 ± 16.39 (L), 106.31 ± 16.32 (R)
	MD: 88/103–85% (AE), 71/103–69% (UE)	MD: 95.74 ± 51.06°/s (AE), 88.57 ± 44.07°/s (UE)		MD: 95.90 ± 18.90 (AE), 98.31 ± 20.24 (UE)
Du Yi et al. 2021 [42]	VM: 18% *			
	MD: 60% *			
ElSherif M et al. 2018 [43]	VM: 21/80–26.3%	VM: >50°/s	VM: 100–250 ms	
	HC: 3/40–7.5%	HC: >50°/s	HC: 100–250 ms	
ElSherif M et al. 2020 [44]	VM: 9/25–36%			
	HC: 2/20–10%			
Kaçan M et al. 2023 [39]	VM: 1/20			
	MD: 3/20			
Yilmaz MS et al. 2021 [37]	VM: 5/50–10%			
	(overt 8%, covert 2%)			
	MD: 22/59–37.3%			
	(overt 30.5%, covert 6.8%) (AE)			
Yollu U et al. 2017 [14]	VM: 11/21–52.4%			
	(overt 23.8%, covert 33.3%)			
	HC: 3/20–15%			
	(overt 0%, covert 15%)			

Abbreviations: AE: affected ears; HC: healthy controls; L: left ear; R: Right ear; UE: unaffected ears. * The saccade rate in this study was determined by the saccade divergence: PR score appearance rate on the two ears. ** According to Du et al. [40], head impulses end at 140 ms, and they do not use subtraction in the saccade latency time measurements they provide. On the other hand, ElSherif et al. most probably use subtraction in the relative data they provide, but they do not analyze it further [35].

## Data Availability

The original contributions presented in this study are included in the article/Supplementary Material. Further inquiries can be directed to the corresponding author.

## Supplementary Materials

The following supporting information can be downloaded at https://www.mdpi.com/article/10.3390/audiolres16010001/s1, Table S1: Bias assessment for the included studies.

## References

[1] Blödow A., Heinze M., Bloching M.B., von Brevern M., Radtke A., Lempert T. (2014). Caloric Stimulation and Video-Head Impulse Testing in Ménière’s Disease and Vestibular Migraine. Acta Oto-Laryngol..

[2] Salt A.N., Plontke S.K. (2010). Endolymphatic Hydrops: Pathophysiology and Experimental Models. Otolaryngol. Clin. N. Am..

[3] Kharkheli E., Japaridze S., Kevanishvili Z., Oz I., Ozluoglu L.N. (2019). Correlation between Vestibular Evoked Myogenic Potentials and Disease Progression in Ménière’s Disease. ORL.

[4] Eggermont J.J. (2017). Ups and Downs in 75 Years of Electrocochleography. Front. Syst. Neurosci..

[5] Lin M.Y., Timmer F.C., Oriel B.S., Zhou G., Guinan J.J., Kujawa S.G., Herrmann B.S., Merchant S.N., Rauch S.D. (2006). Vestibular Evoked Myogenic Potentials (VEMP) Can Detect Asymptomatic Saccular Hydrops. Laryngoscope.

[6] Grosser D., Willenborg K., Dellani P., Avallone E., Götz F., Böthig D., Warnecke A., Lanfermann H., Lenarz T., Giesemann A. (2021). Vestibular Aqueduct Size Correlates with the Degree of Cochlear Hydrops in Patients with and without Menière’s Disease. Otol. Neurotol..

[7] Tsilivigkos C., Vitkos E.N., Ferekidis E., Warnecke A. (2024). Can Multifrequency Tympanometry Be Used in the Diagnosis of Meniere’s Disease? A Systematic Review and Meta-Analysis. J. Clin. Med..

[8] Cha Y.H., Brodsky J., Ishiyama G., Sabatti C., Baloh R.W. (2007). The Relevance of Migraine in Patients with Meńière’s Disease. Acta Oto-Laryngol..

[9] Frejo L., Martin-Sanz E., Teggi R., Trinidad G., Soto-Varela A., Santos-Perez S., Manrique R., Perez N., Aran I., Almeida-Branco M.S. (2017). Extended Phenotype and Clinical Subgroups in Unilateral Meniere Disease: A Cross-Sectional Study with Cluster Analysis. Clin. Otolaryngol..

[10] Tajti J., Párdutz Á., Vámos E., Tuka B., Kuris A., Bohár Z., Fejes A., Toldi J., Vécsei L. (2011). Migraine Is a Neuronal Disease. J. Neural Transm..

[11] Seemungal B., Kaski D., Lopez-Escamez J.A. (2015). Early Diagnosis and Management of Acute Vertigo from Vestibular Migraine and Ménière’s Disease. Neurol. Clin..

[12] Chang J.C., Brennan K.C., He D., Huang H., Miura R.M., Wilson P.L., Wylie J.J. (2013). A Mathematical Model of the Metabolic and Perfusion Effects on Cortical Spreading Depression. PLoS ONE.

[13] Espinosa-Sanchez J.M., Lopez-Escamez J.A. (2015). New Insights into Pathophysiology of Vestibular Migraine. Front. Neurol..

[14] Yollu U., Uluduz D.U., Yilmaz M., Yener H.M., Akil F., Kuzu B., Kara E., Hayir D., Ceylan D., Korkut N. (2017). Vestibular Migraine Screening in a Migraine-Diagnosed Patient Population, and Assessment of Vestibulocochlear Function. Clin. Otolaryngol..

[15] Lempert T., von Brevern M. (2019). Vestibular Migraine. Neurol. Clin..

[16] Radtke A., von Brevern M., Neuhauser H., Hottenrott T., Lempert T. (2012). Vestibular Migraine: Long-Term Follow-Up of Clinical Symptoms and Vestibulocochlear Findings. Neurology.

[17] Dieterich M., Obermann M., Celebisoy N. (2016). Vestibular Migraine: The Most Frequent Entity of Episodic Vertigo. J. Neurol..

[18] Neuhauser H., Lempert T. (2004). Vertigo and Dizziness Related to Migraine: A Diagnostic Challenge. Cephalalgia.

[19] von Brevern M., Radtke A., Clarke A.H., Lempert T. (2004). Migrainous Vertigo Presenting as Episodic Positional Vertigo. Neurology.

[20] Neff B.A., Staab J.P., Eggers S.D., Carlson M.L., Schmitt W.R., Van Abel K.M., Worthington D.K., Beatty C.W., Driscoll C.L., Shepard N.T. (2012). Auditory and Vestibular Symptoms and Chronic Subjective Dizziness in Patients with Ménière’s Disease, Vestibular Migraine, and Ménière’s Disease with Concomitant Vestibular Migraine. Otol. Neurotol..

[21] Lempert T., Olesen J., Furman J., Waterston J., Seemungal B., Carey J., Bisdorff A., Versino M., Evers S., Newman-Toker D. (2012). Vestibular Migraine: Diagnostic Criteria. J. Vestib. Res..

[22] Lopez-Escamez J.A., Carey J., Chung W.H., Goebel J.A., Magnusson M., Mandalà M., Newman-Toker D.E., Strupp M., Suzuki M., Trabalzini F. (2015). Diagnostic Criteria for Menière’s Disease. J. Vestib. Res..

[23] Halmagyi G.M., Chen L., MacDougall H.G., Weber K.P., McGarvie L.A., Curthoys I.S. (2017). The Video Head Impulse Test. Front. Neurol..

[24] Page M.J., McKenzie J.E., Bossuyt P.M., Boutron I., Hoffmann T.C., Mulrow C.D., Shamseer L., Tetzlaff J.M., Akl E.A., Brennan S.E. (2021). The PRISMA 2020 Statement: An Updated Guideline for Reporting Systematic Reviews. BMJ.

[25] Wohlin C. Guidelines for Snowballing in Systematic Literature Studies and a Replication in Software Engineering. Proceedings of the 18th International Conference on Evaluation and Assessment in Software Engineering.

[26] Downes M.J., Brennan M.L., Williams H.C., Dean R.S. (2016). Development of a Critical Appraisal Tool to Assess the Quality of Cross-Sectional Studies (AXIS). BMJ Open.

[27] Cohen H.S., Stitz J., Sangi-Haghpeykar H., Williams S.P., Mulavara A.P., Peters B.T., Bloomberg J.J. (2018). Utility of Quick Oculomotor Tests for Screening the Vestibular System in the Subacute and Chronic Populations. Acta Oto-Laryngol..

[28] Kim H.S., Oh E.H., Kim J.Y., Choi S.Y., Choi K.D., Choi J.H. (2022). Discordant Vestibulo-Ocular Reflex Function According to the Frequency and Mode of Stimulation. J. Neurol..

[29] Gerb J., Becker-Bense S., Zwergal A., Huppert D. (2022). Vestibular Syndromes after COVID-19 Vaccination: A Prospective Cohort Study. Eur. J. Neurol..

[30] Eza-Nuñez P., Fariñas-Alvarez C., Fernandez N.P. (2016). Comparison of Three Diagnostic Tests in Detecting Vestibular Deficit in Patients with Peripheral Vestibulopathy. J. Laryngol. Otol..

[31] Wu Y., Ling X., Song N., Yan S., Wang W., Yang X., Gu P. (2023). Comparison of Clinical Characteristics and Vestibular Function Test Results in Patients with Vestibular Migraine and Menière’s Disease. Braz. J. Otorhinolaryngol..

[32] Kim Y., Kang B.C., Yoo M.H., Park H.J. (2022). Differential Involvement of Lateral Semicircular Canal and Otolith Organs in Common Vestibular Disorders. Front. Neurol..

[33] Hannigan I.P., Welgampola M.S., Watson S.R.D. (2021). Dissociation of Caloric and Head Impulse Tests: A Marker of Meniere’s Disease. J. Neurol..

[34] Waissbluth S., Sepúlveda V., Leung J.S., Oyarzún J. (2022). Caloric and Video Head Impulse Test Dissociated Results in Dizzy Patients. Front. Neurol..

[35] Nishikawa D., Wada Y., Shiozaki T., Shugyo M., Ito T., Ota I., Kitahara T. (2021). Patients with Vertigo/Dizziness of Unknown Origin during Follow-Ups by General Otolaryngologists at Outpatient Town Clinic. Auris Nasus Larynx.

[36] Salmito M.C., Ganança F.F. (2021). Video Head Impulse Test in Vestibular Migraine. Braz. J. Otorhinolaryngol..

[37] Yilmaz M.S., Egilmez O.K., Kara A., Guven M., Demir D., Genc Elden S. (2021). Comparison of the Results of Caloric and Video Head Impulse Tests in Patients with Meniere’s Disease and Vestibular Migraine. Eur. Arch. Oto-Rhino-Laryngol..

[38] Balayeva F., Kirazlı G., Celebisoy N. (2023). Vestibular Test Results in Patients with Vestibular Migraine and Meniere’s Disease. Acta Oto-Laryngol..

[39] Kaçan M., Kirazlı G., Balayeva F., Celebisoy N. (2023). Recurrent Vestibulopathy: Comparison of Vestibular Test Results with Ménière’s Disease and Vestibular Migraine. Audiol. Neurotol..

[40] Du Y., Liu X., Ren L., Wang Y., Wu Z. (2022). Analysis of Video Head Impulse Test Saccades Data in Patients with Vestibular Migraine or Probable Vestibular Migraine. J. Otol..

[41] Du Y., Liu X., Ren L., Wang Y., Ji F., Guo W., Wu Z. (2023). Saccades of Video Head Impulse Test in Meniere’s Disease and Vestibular Migraine: What Can We Learn from?. J. Otol..

[42] Du Y., Ren L., Liu X., Guo W., Wu Z., Yang S. (2021). The Characteristics of vHIT Gain and PR Score in Peripheral Vestibular Disorders. Acta Oto-Laryngol..

[43] ElSherif M., Reda M.I., Saadallah H., Mourad M. (2018). Video Head Impulse Test (vHIT) in Migraine Dizziness. J. Otol..

[44] ElSherif M., Reda M.I., Saadallah H., Mourad M. (2020). Eye Movements and Imaging in Vestibular Migraine. Acta Otorrinolaringol. Esp..

[45] Martines F., Dispenza F., Montalbano C., Priola R., Torrente A., La Gumina R., Brighina F., Galletti F., Salvago P. (2020). Comparison of Electrocochleography and Video Head Impulse Test Findings in Vestibular Migraine and Ménière Disease: A Preliminary Study. J. Int. Adv. Otol..

[46] American Academy of Otolaryngology-Head and Neck Foundation, Inc (1995). Committee on Hearing and Equilibrium guidelines for the diagnosis and evaluation of therapy in Menière’s disease. Otolaryngol. Head Neck Surg..

[47] MacDougall H.G., Weber K.P., McGarvie L.A., Halmagyi G.M., Curthoys I.S. (2009). The Video Head Impulse Test: Diagnostic Accuracy in Peripheral Vestibulopathy. Neurology.

[48] Lasheen R.M., Kolkaila E.A., Elmehalawy T.H., Mady N., Nada N. (2024). Video Head Impulse Test (vHIT) Unravels the Hidden Pathology in Chronic Vestibular Deficit. Egypt. J. Otolaryngol..

[49] Bronstein A.M., Patel M., Arshad Q. (2015). A Brief Review of the Clinical Anatomy of the Vestibular-Ocular Connections—How Much Do We Know?. Eye.

[50] Elkawy F.S.A., Elgohary M.A.E., Beheiry R.M. (2024). Video Head Impulse Test (vHIT) versus Videonystagmography (VNG) in Migraine with Dizziness. Egypt. J. Otolaryngol..

[51] McGarvie L.A., Curthoys I.S., MacDougall H.G., Halmagyi G.M. (2015). What Does the Head Impulse Test Versus Caloric Dissociation Reveal about Vestibular Dysfunction in Ménière’s Disease?. Ann. N. Y. Acad. Sci..

[52] Bartolomeo M., Biboulet R., Pierre G., Mondain M., Uziel A., Venail F. (2014). Value of the Video Head Impulse Test in Assessing Vestibular Deficits Following Vestibular Neuritis. Eur. Arch. Oto-Rhino-Laryngol..

[53] Zellhuber S., Mahringer A., Rambold H.A. (2014). Relation of Video-Head-Impulse Test and Caloric Irrigation: A Study on the Recovery in Unilateral Vestibular Neuritis. Eur. Arch. Oto-Rhino-Laryngol..

[54] Mavrodiev V., Strupp M., Vinck A.S., van de Berg R., Lehner L. (2024). The Dissociation Between Pathological Caloric Testing and a Normal Video Head Impulse Test Helps Differentiate Between Menière’s Disease, Vestibular Migraine, and Other Vestibular Disorders: A Confirmatory Study in a Large Cohort of 2101 Patients. Front. Neurol..

[55] Leigh R.J., Zee D.S. (2015). The Neurology of Eye Movements.

[56] de Brouwer S., Missal M., Barnes G., Lefèvre P. (2002). Quantitative Analysis of Catch-Up Saccades During Sustained Pursuit. J. Neurophysiol..

[57] Van Nechel C., Bostan A., Duquesne U., Hautefort C., Toupet M. (2019). Visual Input Is the Main Trigger and Parametric Determinant for Catch-Up Saccades During Video Head Impulse Test in Bilateral Vestibular Loss. Front. Neurol..

[58] Anson E.R., Bigelow R.T., Carey J.P., Xue Q.L., Studenski S., Schubert M.C., Weber K.P., Agrawal Y. (2016). Aging Increases Compensatory Saccade Amplitude in the Video Head Impulse Test. Front. Neurol..

[59] Rey-Martinez J., Batuecas-Caletrio A., Matiño E., Perez Fernandez N. (2015). HITCal: A Software Tool for Analysis of Video Head Impulse Test Responses. Acta Oto-Laryngol..

