# Supplementary material for: The Use of vHIT in the Differential Diagnosis Between Vestibular Migraine and Meniere’s Disease: A Systematic Review and Meta-Analysis
Source: Audiol Res. 2025 Dec 19;16(1):1. doi: 10.3390/audiolres16010001 (PMC12821388; doi:10.3390/audiolres16010001)
Supplement: Supplementary file 1 [file audiolres-16-00001-s001.zip › audiolres-4051875-supplementary.pdf]

	Q1	Q2	Q3	Q4	Q5	Q6	Q7	Q8	Q9	Q10	Q11	Q12	Q13	Q14	Q15	Q16	Q17	Q18	Q19	Q20
Balayeva F et al. 2023 [38]	Yes	Yes	Yes	Yes	Yes	DK(unclear if consecutive or random sampling was used to avoid selection bias)	No	Yes	Yes	Yes	Yes	Yes	No	No	Yes	Yes	Yes	Yes	No	Yes
Blödow A et al. 2014 [1]	Yes	Yes	No	Yes	Yes	No	No	Yes	Yes	Yes	Yes	Yes	Yes	No	Yes	Yes	Yes	No	DK (funding sources not discussed)	Yes
Du Yi et al. 2022 [40]	Yes	Yes	No	Yes	Yes	No	No	Yes	Yes	Yes	Yes	Yes	Yes	No	Yes	Yes	Yes	No	No	Yes
Du Yi et al. 2023 [41]	Yes	Yes	No	Yes	Yes	No	No	Yes	Yes	Yes	Yes	Yes	Yes	No	Yes	Yes	Yes	No	No	Yes
Du Yi et al. 2021 [42]	Yes	Yes	No	Yes	Yes	No	No	Yes	Yes	Yes	Yes	Yes	DK (no mention of response rate)	No	Yes	Yes	Yes	No	No	Yes
ElSherif M et al. 2018 [43]	Yes	Yes	No	Yes	Yes	No	No	Yes	Yes	Yes	yes	Yes	DK (no mention of response rate)	No	Yes	Yes	Yes	No	DK (not mentioned in the text)	Yes
ElSherif M et al. 2020 [44]	Yes	Yes	No	Yes	Yes	No	No	Yes	Yes	Yes	Yes	Yes	DK (no mention of response rate)	No	Yes	Yes	Yes	No	No	Yes

Kaçan M et al. 2023 [39]	Yes	Yes	Yes	Yes	Yes	No	No	Yes	Yes	Yes	Yes	Yes	DK (no mention of response rate)	No	Yes	Yes	Yes	No	No	Yes
Martines F et al. 2020 [45]	Yes	Yes	No	Yes	Yes	No	No	Yes	Yes	Yes	Yes	Yes	DK (no mention of response rate)	No	Yes	Yes	Yes	Yes	No	Yes
Yilmaz MS et al. 2021 [37]	Yes	Yes	Yes	Yes	Yes	No	No	Yes	Yes	Yes	Yes	Yes	DK (no mention of response rate)	No	Yes	Yes	Yes	No	No	Yes
Yollu U et al. 2017 [14]	Yes	Yes	No	Yes	Yes	No	No	Yes	Yes	Yes	Yes	Yes	DK (no mention of response rate)	No	Yes	Yes	Yes	Yes	No	Yes

Table S1. Bias assessment for the included studies.
Abbreviations: DK: Don’t know
